# Phenotypic plasticity in reproductive effort: malaria parasites respond to resource availability

**DOI:** 10.1098/rspb.2017.1229

**Published:** 2017-08-02

**Authors:** Philip L. G. Birget, Charlotte Repton, Aidan J. O'Donnell, Petra Schneider, Sarah E. Reece

**Affiliations:** Institutes of Evolutionary Biology, and Immunology and Infection Research, University of Edinburgh, Edinburgh EH9 3FL, UK

**Keywords:** phenotypic plasticity, genetic variation, life-history strategy, gametocytes, reticulocytes, resource allocation trade-off

## Abstract

The trade-off between survival and reproduction is fundamental in the life history of all sexually reproducing organisms. This includes malaria parasites, which rely on asexually replicating stages for within-host survival and on sexually reproducing stages (gametocytes) for between-host transmission. The proportion of asexual stages that form gametocytes (reproductive effort) varies during infections—i.e. is phenotypically plastic—in response to changes in a number of within-host factors, including anaemia. However, how the density and age structure of red blood cell (RBC) resources shape plasticity in reproductive effort and impacts upon parasite fitness is controversial. Here, we examine how and why the rodent malaria parasite *Plasmodium chabaudi* alters its reproductive effort in response to experimental perturbations of the density and age structure of RBCs. We show that all four of the genotypes studied increase reproductive effort when the proportion of RBCs that are immature is elevated during host anaemia, and that the responses of the genotypes differ. We propose that anaemia (counterintuitively) generates a resource-rich environment in which parasites can afford to allocate more energy to reproduction (i.e. transmission) and that anaemia also exposes genetic variation to selection. From an applied perspective, adaptive plasticity in parasite reproductive effort could explain the maintenance of genetic variation for virulence and why anaemia is often observed as a risk factor for transmission in human infections.

## 1. Introduction

Parasites are exposed to rapid and extensive variation in the environmental conditions they experience inside their hosts and vectors, both during infections and across different hosts (e.g. dynamic immune responses, competition with other parasites, fluctuations in the availability and quality of resources). Therefore, parasites, like any organism which experiences frequent environmental change, could use phenotypic plasticity—the ability to match phenotypes to different environmental conditions—to maintain their fitness in the face of changing conditions [1,2]. However, because parasites have traditionally been viewed as organisms with inflexible strategies, their environmental sensing mechanisms are assumed to be directed towards maintaining homeostasis [3]. Thus, adaptive plasticity in parasites has generally been overlooked in evolutionary biology and is controversial in applied bioscience [4,5]. For malaria parasites (*Plasmodium* spp.), there is mounting evidence that allocation to within-host growth versus between-host transmission is phenotypically plastic [6,7]. Malaria parasites rely on asexually replicating stages for within-host survival and on sexually reproducing stages (gametocytes) for between-host transmission. Therefore, for malaria and all other parasites that use distinct stages for transmission and within-host replication (e.g. trypanosomes), between-host transmission is equivalent to ‘reproduction’ of an infection and within-host replication determines the ‘survival’ of an infection [6,8,9]. The proportion of asexual stages in each cycle of replication that commit to forming gametocytes represents the parasite's reproductive effort (called the ‘conversion rate’ in parasitology). Why conversion rates are generally low and highly variable in malaria parasites are long-standing questions in parasitology [6,10] and explaining plasticity in reproductive effort is a major aim of evolutionary biology [11–13].

The fundamental life-history trade-off between survival and reproduction has attracted much theoretical and empirical attention [14–17]. The consequences of diverting energetic resources from maintenance of the organism into reproduction means that organisms must balance their current reproductive effort against their prospects for survival and future reproduction. For most organisms, a reproductive event comes with several types of costs (e.g. egg production, parental care, competition for mates), many of which can affect an organism's survival [18]. For example, breeding-associated immobility or the need for increased foraging to feed offspring can expose parents to increased predation risk [19,20]. For malaria parasites, allocating cells to become gametocytes comes at the instant cost of a reduced number of asexual stages, needed to perpetuate the infection. Theory predicts that, compared with older organisms, whose residual reproductive value (RRV, the age-specific expectation of future offspring) is lower, young organisms should allocate less into current reproduction (‘reproductive restraint’) so as to minimize risk to their survival prospects [15]. By contrast, reproductive effort should increase as the probability of future reproductive success decreases [13], and in the last reproductive event of their life, i.e. when the RRV is very small, organisms should allocate all of their remaining energy to reproduction (‘terminal investment’).

In addition to the age of the organism, the current environment and an organism's physiological characteristics are also expected to affect reproductive effort. Accounting for these effects, summarized as ‘state variables’, has given rise to theory that complements age-based life-history theory [17,21–23]. For example, terminal investment may not just be observed when age is the main reason for an organism's death, but also if external factors cause the probability of future reproduction to be near zero [24]. Conversely, if current conditions are not conducive to reproduction (e.g. resources are limited), an organism may be selected to exert reproductive restraint and delay reproduction until environmental conditions improve [25–27]. Finally, state-based and age-based life history interact because physiological state varies over an organism's lifetime [11,28]. In summary, an optimally behaving organism is expected to adjust its reproductive effort over successive bouts of reproduction according to interactions between environmental conditions, energetic reserves and expected lifespan. The predictions of age- and state-based theories are supported by empirical data from diverse laboratory models and natural systems [15,16,29–32], confirming the fitness benefits of adjusting reproductive effort in relation to circumstances.

**Figure 1.**
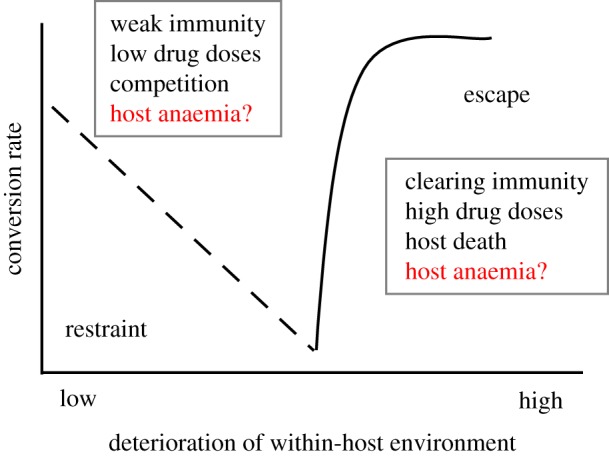
Cartoon of a reaction norm for conversion rate against conditions experienced by parasites inside the mammalian host, adapted from [6]. Importantly, the *x*-axis does not represent time since infection, but a given stress, or combination of stressors, experienced at any point during the infection that reduces the condition/state of parasites. The dotted line represents a decrease in conversion rate (‘restraint’) as the parasites experience a loss of condition/state, but the exact functional form of this is not known. If the within-host environment has deteriorated substantially and recovery of condition/state is unlikely or impossible parasites should make a terminal investment by putting all resources into transmission (‘escape’). Factors in the boxes denote circumstances thought to induce reproductive restraint or terminal investment, but the effect of variable red blood cell resources during anaemia is unclear. (Online version in colour.)

Life-history theory has also been applied to explain plasticity in the reproductive effort of malaria parasites, which appear to adjust conversion rates in response to information about their within-host environment and the density of clone-mates within the host [33–39]. A clonal parasite population inside a host is the selective equivalent of a single organism [40] and can experience considerable variation in within-host environments during infections and in different hosts. Characteristics of the in-host environment can affect state, which for malaria parasites is associated with asexually replicating stages because they make up the bulk of parasite biomass and produce gametocytes. Environmental conditions thought to affect parasite state are immune attack, drug treatment, competition with con specific parasite strains and a variable supply of red blood cells (RBCs) [6]. Parasites should therefore prioritize allocation to asexual stages to maintain the infection when faced with a moderate loss of state because prolonging in-host survival rewards parasites with future transmission opportunities [7,34,41,42]. By contrast, parasites should allocate all remaining resources to transmission when faced with a situation where in-host survival is unlikely (e.g. clearance by drugs or strong immunity) [6,37,38,43] or when the infection is likely to end due to host death. Finally, an increase in state (e.g. due to an enrichment of the environment) allows parasites to allocate more to gametocytes, as within-host survival is not at risk. These strategies can be represented in a reaction norm for conversion rate which illustrates how the circumstances that parasites find themselves in determine the allocation made to gametocytes at each cycle of replication (figure 1).

Mounting evidence suggests that malaria parasites have evolved to adjust conversion rates according to circumstances—including changes in the supply of an essential resource—RBCs [35,44–46]. Most studies report an increase in gametocyte densities when hosts become anaemic. Only two studies link elevated gametocyte densities to higher conversion in response to anaemia [35,45], and most findings are based on post hoc correlations of observational data in which confounding changes in asexual densities cannot be accounted for [44,46,47]. Moreover, it is not known whether increased conversion occurs because parasites are making a terminal investment (e.g. due to RBC limitation or because an anaemic host has a high mortality risk), or because the in-host environment has improved and parasites can afford to reproduce. These contradictory hypotheses emerge because anaemia is complex. The population of circulating RBCs within the host is characterized quantitatively by their overall density (i.e. number of RBCs per millilitre blood) and qualitatively by their age structure (the frequency of immature RBCs, termed reticulocytes, versus mature RBCs, termed normocytes). The density and/or frequency of reticulocytes and normocytes matters because different parasite species preferentially invade different age classes [6,48]. A further complication arises because parasites may not be selected to respond to anaemia *per se*, but simply use it as a proxy for the appearance of immune responses that have a major effect on state [49–51].

Here, we avoid confounding changes in immune responses and variation in asexual density to ask how parasites adjust conversion rates in response to the in-host environmental characteristic anaemia. Our approach enables us to examine several different parasite genotypes of the rodent malaria parasite *Plasmodium chabaudi* to assess genetic and genotype-by-environment influences on conversion rate. We find that all genotypes increase their conversion in response to anaemic conditions and that different genotypes do so to different extents. Further analysis suggests that parasites respond to the frequency of reticulocytes versus normocytes and that parasites increase conversion because they are taking advantage of an increase in resources rather than making a terminal investment.

## 2. Material and methods

We pre-treated hosts with different doses of a drug that modifies the age structure (measured as the proportion of reticulocytes) and overall density of RBCs in the blood (phenylhydrazine, PHZ), then inoculated mice with parasites of one of several genotypes. We then measured the density of asexual parasites and gametocytes to infer the conversion rate.

### (a) Parasites and hosts

We obtained C57BL/6 female mice (aged six to eight weeks) in-house (University of Edinburgh) and *P. chabaudi* clones AJ, AS, CR and ER from the Edinburgh Malaria Reagent Repository (University of Edinburgh). *Plasmodium chabaudi* was isolated between 1948 and 1974 from African thicket rats, *Thamnomys* spp., in Central Africa [52]. After cloning, the parasite genotypes have been cryopreserved and undergone regular transmission through mosquitoes to maintain their wild-type phenotypes [53]. The four *P. chabaudi* genotypes chosen span the diversity of conversion rates and virulence reported from previous experiments [34,54,55]. Four days after PHZ treatment (see below), hosts were infected intravenously with 5 × 10^6^–1 × 10^7^ parasitized RBCs.

### (b) Perturbing red blood cell resources in the within-host environment

We used PHZ to generate different RBC resource environments in the blood. Phenylhydrazine causes the clearance of circulating RBCs [56] and the resulting anaemia stimulates the release of immature RBCs (reticulocytes) from the bone marrow. We found in a pilot study that injecting 120, 30 and 0 mg kg^−1^ (control) of PHZ generated non-overlapping environments in terms of both total RBC density and reticulocyte proportion (see electronic supplementary material, figures S1 and S2) 2 days after injection, and that these differences persisted for 5 more days (see the electronic supplementary material). The variation in total RBC density and reticulocyte proportion observed for these doses corresponds to environments typically encountered by parasites during infection: the control treatment emulates the initial environment encountered in a healthy host, the 120 mg kg^−1^ treatment reflects conditions after the peak of infection when hosts are most sick, whereas the 30 mg kg^−1^ treatment resembles the RBC environment in a recovering host.

### (c) Experimental design and data collection

Four days before infection (day -4 post infection, PI) we injected mice with 120 mg kg^−1^ (*n* = 23 mice), 30 mg kg^−1^ (*n* = 23) or 0 mg kg^−1^ (control treatment, *n* = 22) PHZ and on day 0 PI we infected each mouse with one of the four clones. This gave us a fully cross-factored design with 12 different treatment combinations (4 genotypes × 3 PHZ treatments). From day 1 PI to day 3 PI, we monitored mice daily at 09.00 by taking 2 μl of blood to quantify RBC density [57], making a thin blood smear, and collecting 10 μl blood for RT-qPCR to quantify gametocytes approximately aged 35 hours or older (since bursting from a committed schizont) [42]. RNA extraction was performed as described in [58] with minor changes to the protocol (see the electronic supplementary material). We deliberately focused on the early infection dynamics to maximize the likelihood of observing parasite responses to the RBC environments they encountered upon infection, rather than confounding factors that develop as infections progress (e.g. immune responses, divergence in parasite densities between treatments). Blood smears were used to estimate the density of asexual parasites and the proportion of RBCs that were reticulocytes or normocytes. All host measures relating to the RBC environment were inferred from the age structure and total density of RBCs; the density of reticulocytes was estimated as the proportion of reticulocytes multiplied by the total RBC density and the density of normocytes as (1−the proportion of reticulocytes), multiplied by total RBC density.

### (d) Estimation of conversion rate and statistical analysis

Data were analysed using R v. 3.0.2. We used ANOVAs to assess the effect of PHZ on the within-host environment and on asexual parasite density. We then carried out two analyses to ask how anaemia affects conversion rates. Using ANCOVAs, we first tested the effect of genotype and PHZ treatment on conversion while also controlling for asexual density. All test statistics, degrees of freedom and *p*-values reported are from maximum-likelihood-based deletion tests (i.e. comparing a model with and without the explanatory variable of interest) [59]. Second, we fitted four ANCOVA models that decomposed the effects of PHZ into the densities and frequencies of RBC types to identify which in-host environmental variables correlate most closely with conversion rates. These models were simplified using maximum-likelihood-based deletion tests as above and the minimal models were compared by AIC. To infer conversion rate, we compared the summed gametocyte densities on days 2 and 3 PI to the summed density of their source populations of asexual parasites whose densities were recorded on days 1 and 2 PI. In the absence of differences in asexual parasite density, analysing pooled gametocyte densities directly avoids the difficulties of accurately calculating conversion rate using PCR data [6,60]. Gametocyte densities on days 2 and 3 PI may include gametocytes produced in donor mice. However, as all mice within each clone group were infected from the same donor mouse and the number of gametocytes or sexually committed parasites in inocula is small, this is unlikely to influence differences between treatments.

## 3. Results

### (a) Modifying resources in the within-host environment

**Figure 2.**
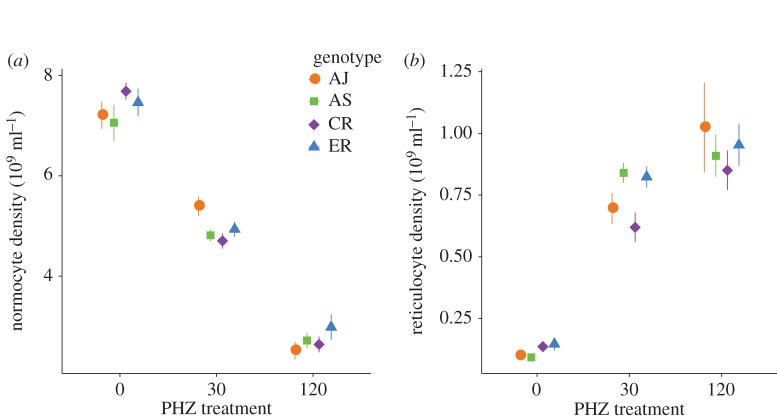
Mean ± standard error of the mean (SE) normocyte density (*a*) and reticulocyte density (*b*) on days 0–2 PI (*n* = 68) by PHZ treatment (0, 30, 120 mg kg^−1^) and genotype. Normocyte and reticulocyte densities are significantly different between PHZ treatments but not between genotypes. (Online version in colour.)

For the duration of experimental infections, hosts in the different PHZ treatment groups had significantly different total RBC densities and age structures (figure 2; electronic supplementary material, table S1). As expected, normocyte density was reduced by PHZ in a dose-dependent manner, whereas the density and proportion of reticulocytes varied in the reverse fashion (figure 2). There are no genotype or genotype by PHZ treatment effects, i.e. within a given PHZ treatment, all genotypes encountered the same RBC environment (electronic supplementary material, table S1). The mean total RBC density from all genotypes combined (expressed as 10^9^ cells ml^−1^) from day 0 PI to day 2 PI varied from 3.03(±0.18) to 4.57(±0.17) in the 120 mg kg^−1^ treatment, from 5.63(±0.14) to 5.68(±0.13) in the 30 mg kg^−1^ treatment and from 7.04(±0.34) to 7.71(±0.15) in the control treatment. The proportion of reticulocytes varied from 0.21(±0.02) to 0.27(±0.001) in the 120 mg kg^−1^ treatment, from 0.12(±0.01) to 0.13(±0.008) in the 30 mg kg^−1^ treatment and from 0.01(±0.002) to 0.02(±0.002) in the control treatment.

### (b) Conversion rate in perturbed within-host environments

**Figure 3.**
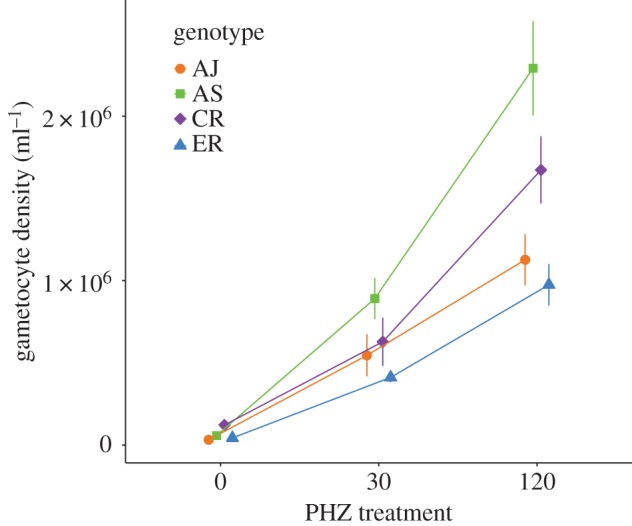
Reaction norms for conversion rate (mean ± SE gametocyte density for days 2 and 3 PI) of four genotypes across PHZ treatments (left to right along *x*-axis represents a decrease in total red blood cell density and an increase in reticulocyte proportion). All genotypes increase their conversion as PHZ dose increases, but to different extents (note, there is no significant difference between AJ and ER). The points are dodged horizontally for clarity. (Online version in colour.)

Asexual density increases from day 1 to day 2 PI but there is no significant difference in their summed densities between genotypes within each PHZ treatment (*F*(3, 62) = 1.62, *p* = 0.193; electronic supplementary material, figure S3) or between PHZ treatments (*F*(2, 65) = 2.81, *p* = 0.067; electronic supplementary material, table S2). Thus, the summed gametocyte densities on days 2 and 3 PI reflect the conversion decision taken by asexuals on days 1 and 2 PI. However, we also control for asexual density in the following statistical models to account for any subtle variation between individual infections. The mean total gametocyte densities (over days 2 and 3 PI) vary significantly across PHZ treatments and genotypes. All genotypes increase their gametocyte densities (0 mg kg^−1^ < 30 mg kg^−1^ < 120 mg kg^−1^) with increasing PHZ dose, but they vary in the magnitude of their responses (figure 3; electronic supplementary material, table S3). Once adjusted for variation in asexual density, the interaction between genotype and PHZ treatment explains 84% of the variance in gametocyte densities. To assess whether each genotype employs a different conversion rate strategy, we tested whether any genotypes could be grouped together without causing significant change in model deviance. The responses of AJ and ER did not differ significantly from each other (*F*(3, 54) = 0.08, *p* = 0.970) but all other genotypes follow significantly different reaction norms. In response to increasing PHZ doses, AS increases its gametocyte density most, followed by CR, and finally AJ/ER. Also, mean pairwise differences between genotypes are significantly greater in PHZ-treated mice, with approximately sixfold greater variation between genotypes expressed in the 120 mg kg^−1^ environment compared with 0 mg kg^−1^ (*F*(2, 15) = 11.47, *p* < 0.001, figure 3).

### (c) Conversion rate and RBC resources

**Figure 4.**
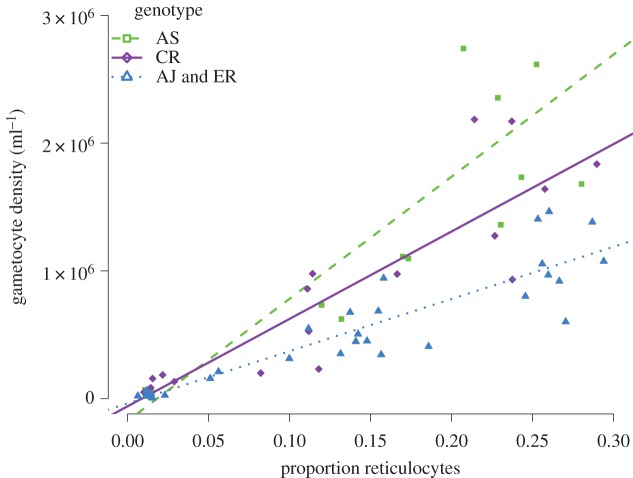
Gametocyte density for each mouse (mean of days 2 and 3 PI) correlates with RBC age structure (the mean proportion of RBCs that are reticulocytes across days 0, 1 and 2 PI for each mouse), and fitted lines illustrate the reaction norms for the different genotypes (note, there is no significant difference between AJ and ER). Data from the control group cluster around the origin (median reticulocyte frequency: 0.014), data from the 30 mg kg^−1^ group span a reticulocyte frequency from 0.06 to 0.19 (median 0.14), and a dose of 120 mg kg^−1^ produced a range between 0.21 and 0.29 (median 0.25). (Online version in colour.)

As PHZ was used only as a means to modify the RBC environment, we expect that parasites do not detect and respond to PHZ itself, but to its effects on the density and/or age structure of RBC resources. As measurements of total RBC density and the proportion of RBCs that are reticulocytes (frequency) are independently determined, we fitted them as explanatory variables (instead of PHZ treatment), together with other variables of interest such as genotype and asexual density, and all their interactions (model 1). Using deletion tests, this model was simplified to the interaction between the frequency of RBC types and genotype (electronic supplementary material, table S4). We then constructed and minimized three other models that differed according to variables inferred from our two independent measurements; reticulocyte density (model 2), normocyte density (model 3) or total RBC density (model 4). Comparison of the resulting four minimal models (using AIC) reveals that the model containing the frequency-by-genotype interaction is the best overall (model 1, electronic supplementary material, table S5). This model explains 81% of the observed variation in gametocyte densities (electronic supplementary material, table S4) and reveals a positive correlation between gametocyte density and the proportion of RBCs that are reticulocytes, whose slope varies between genotypes (AS > CR > AJ = ER; figure 4).

## 4. Discussion

We found that the RBC resource environment, perturbed by PHZ, can explain more than 80% of variation in the conversion rate of malaria parasites: the higher the frequency of reticulocytes, the more parasites allocate to gametocytes. This pattern is broadly evident for all four genotypes examined but some genotypes are more sensitive than others, suggesting there is genetic variation for plasticity in conversion rates. We concentrated our analysis on the first few days of infection before the densities of the decision-making asexual cohorts could diverge between treatments. This avoids issues in previous studies where other variables associated with conversion, such as the density of asexual parasites or an adaptive immune response, covary with anaemia [34,35,45]. Our experiment also helps to elucidate the long-standing question of which cues parasites use for gametocytogenesis [33]: metrics for the density of RBCs, which is the most obvious measure of anaemia, do not correlate as strongly with conversion rate as the proportion of RBCs that are reticulocytes, suggesting that parasites respond to RBC age structure. Because encounter rates between merozoites and host RBC of different ages during invasion are directionally proportional to the frequency, not the density, of RBC types, encounter rates with different RBC ages could be a proximate mechanism for parasites to assess their RBC environment. It remains possible that instead, parasites respond to some unknown correlate of PHZ treatment, but we expect this is unlikely, given the diversity of data reporting positive associations between anaemia and correlates of conversion that do not involve PHZ.

Why do parasites increase their conversion rate in response to an increase in reticulocytes? The first possibility is that the environment has improved because resource availability has increased and parasites are able to allocate to gametocytes without unduly compromising survival inside the host (i.e. relaxing the need for reproductive restraint). Alternatively, parasites may perceive that the risk of the infection being cleared (by resource limitation, an immune response or host death) is sufficiently high that they should make a terminal investment. We favour the former explanation: it is unlikely that parasites interpret an influx of reticulocytes as a lethal situation because we observe that no hosts died during the experiment, no infections were cleared and asexual growth rate over the duration of the experiment was not compromised. At first, it seems counterintuitive that anaemia could translate into an improvement of environmental quality; indeed, host-adaptive explanation for ‘bystander killing’ of uninfected RBCs implies that anaemia should facilitate the clearance of infection [61–64]. However, the erythropoietin (EPO)-mediated feedback during anaemia brings in reticulocytes which could be a cue for an imminent improvement in the environment, although this seems unlikely because the presence of reticulocytes generally also correlates with the appearance of adaptive immunity. Thus, parasites may simply find reticulocytes to be superior cells compared with normocytes. If a high proportion of reticulocytes is beneficial to parasites, then a terminal investment strategy would be maladaptive. There are several lines of evidence suggesting that malaria parasites respond to the differential resource qualities of reticulocytes and normocytes. For parasite species that strongly prefer reticulocytes, like *P. berghei*, an increase in replication rate and conversion rate in response to PHZ treatment has been reported [65,66]. Even though *P. chabaudi* is thought to be a generalist and able to infect a wide range of RBC ages, reticulocytes may be a better resource than normocytes for *P. chabaudi* for several non-mutually exclusive reasons: (i) reticulocytes carry a more diverse set of cell surface receptors to interact with rhoptry proteins on invading merozoites [67,68]; (ii) reticulocytes are a metabolically more diverse resource [69]; (iii) reticulocytes could be particularly well suited for the development of gametocytes because their longer lifespan matches the longer maturation time and lifespan of gametocytes compared to asexuals [70–72]; and (iv) an infected reticulocyte may produce more merozoites than a normocyte [73], possibly due to their larger size or reduced oxidative stress [74]. Such mechanisms may also explain observations of higher growth rates and conversion rates of *P. chabaudi* in mice treated with EPO, which increases reticulocyte frequency without affecting total RBC density [45].

We observed three different reaction norms for conversion rate across the four genotypes that we examined. These genotypes vary in virulence and the most virulent genotypes (AJ, ER) are less plastic than the less harmful genotypes (CR, AS). Virulence is generally regarded as a fitness-related trait for parasites: virulent genotypes are better competitors against conspecifics sharing the host, and are more likely to survive drug treatment and immune responses [75–78]. Thus, it is not clear why genetic variation for virulence is maintained in natural malaria populations [79]. Because gametocyte density is positively correlated to transmission success, our results suggest that genotypes of low virulence could achieve greater transmission when hosts mount an erythropoietic response and so, compensate for the fitness costs of low virulence. If this holds for human malaria parasites, it suggests that co-infections that cause anaemia (e.g. helminth infections, [80]) could select for less virulent malaria genotypes. Further, anaemic patients may require additional transmission-reducing measures because they may be more infectious than their counterparts [44,72].

## Supplementary Material

Supplementary Material

## Data accessibility

All data from this project can be obtained from our website http://reecelab.science or from S.E.R.
