# Supplementary material for: Phenotypic plasticity in reproductive effort: malaria parasites respond to resource availability
Source: Proc Biol Sci. 2017 Aug 2;284(1860):20171229. doi: 10.1098/rspb.2017.1229 (PMC5563815; doi:10.1098/rspb.2017.1229)
Supplement: Supplementary Material [file rspb20171229supp1.pdf]

Supplementary Material: Phenotypic plasticity in reproductive effort: malaria parasites respond to resource availability

Pilot experiment: Modifying the RBC environment.

We first carried out a pilot experiment to determine which doses of phenylhydrazine (PHZ) create statistically distinguishable RBC resource environments in the blood. Phenylhydrazine results in the clearance of circulating red blood cells (Savill et al. 2009) and the resulting anaemia stimulates the release of immature RBC (reticulocytes) from the bone marrow. We injected one of four doses of 30 mg/kg, 60 mg/kg, 120 mg/kg of PHZ dissolved in PBS or 0 mg/kg (control, PBS only), via the peritoneal cavity, into 6 mice for each treatment dose. We monitored both normocyte (mature RBC) and reticulocyte density by flow cytometry and thin blood smears (Coulter Counter, Beckman Coulter) once or twice a day for 14 days (Ferguson et al. 2003). We asked whether the doses of PHZ created different total RBC densities and age structures, when these differences occurred, and how long they persisted. We found that PHZ reduces red blood cell density below the levels of controls in a dose-dependent manner and concomitantly increases the proportion of reticulocytes in the blood (figure S1). We found that 120 mg/kg, 30 mg/kg and 0 mg/kg (control) generated significantly different environments in terms of both total red blood cell density and reticulocyte proportion (figure S2) two days after injection and that these differences between doses persisted for 5 more days. The environment following treatment with a 60 mg/kg dose overlapped with that of other doses and was therefore not included in the main experiment.

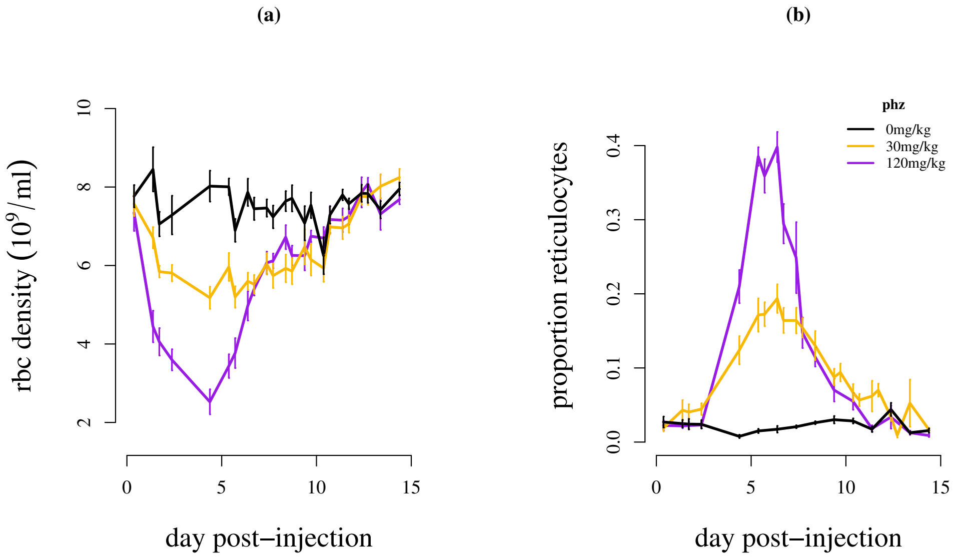

Figure S1. The effect of PHZ doses on total RBC density (a) and the proportion of RBCs that are reticulocytes (b) starting from the day of administration of PHZ (day 0). All mice recover to normal levels after 15 days. Data are the mean and standard error of the mean (SE) for 6 mice per treatment.

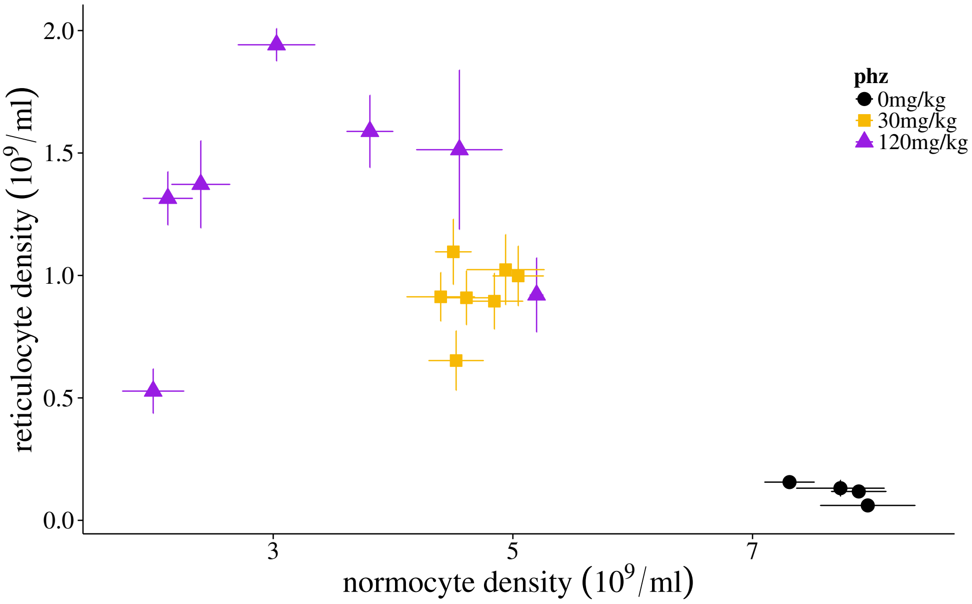

Figure S2. Results of the pilot study to determine which doses of phenylhydrazine (PHZ) create mutually exclusive environments (normocyte density and reticulocyte density) to expose parasites to. Each point corresponds to the mean±SE of all mice in that treatment for one time point post PHZ injection. Only the time points corresponding to day 0 – day 3 post-infection in the main experiment are shown (i.e. days 4–7 post-injection in pilot experiment).

RNA extraction.

We extracted RNA following Schneider et al. (2015) but using the Kingfisher Flex Magnetic Particle Processor (Life Technologies). Additional adjustments were made to the manufacturer's protocol AM1830DW to improve RNA recovery. To ensure full lysis of the blood, 350ul of lysis binding solution and medium mixing speeds were used. Wash steps have been lengthened to 45 seconds and elution to 7 minutes 30 seconds. Minimum temperatures were set to 20°C to prevent variability resulting from low ambient temperatures. All bead transfer steps have been lengthened and an extra bead transfer step was introduced when moving samples from the sample plate and into the elution plate.

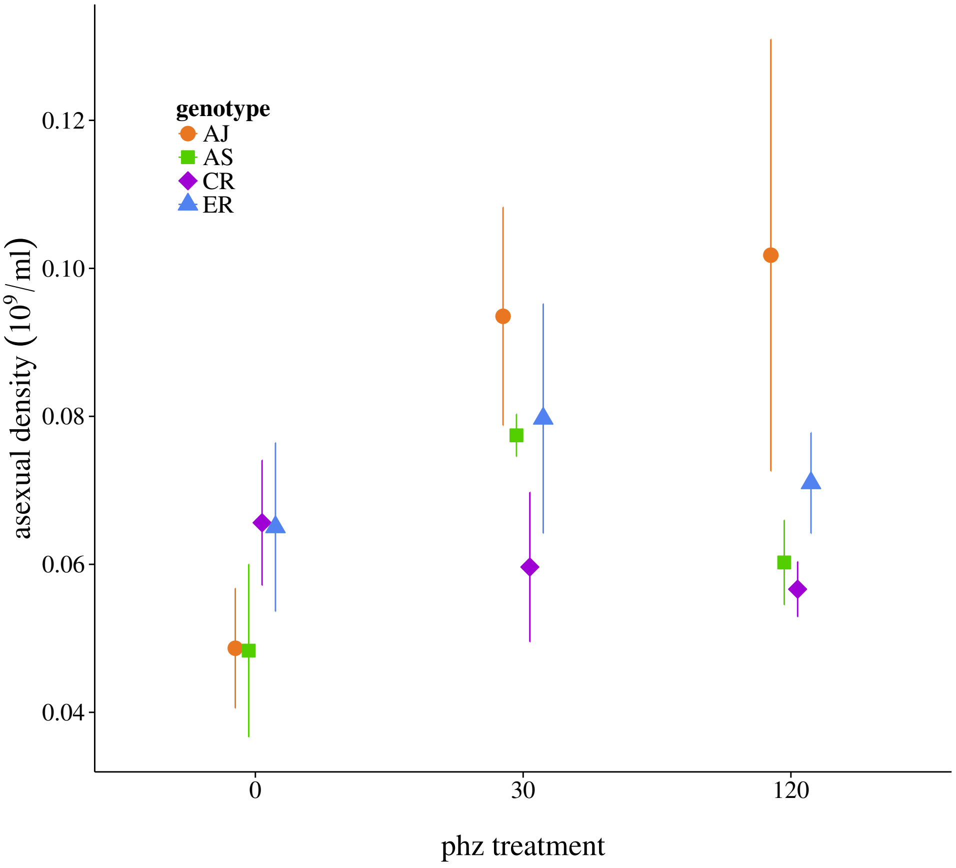

Figure S3. Mean±SE density of the asexual populations (days 1 and 2 post infection) that are the source population for the gametocytes measured, for different genotypes and PHZ treatments.

Table S1. Summary of statistical models examining the effect of PHZ treatment and parasite genotype on the cumulative density (day 0,1,2 PI) of normocytes, reticulocytes and frequency (proportion of those cell types). Frequency was recorded as the proportion of reticulocytes. All models were minimised and test-statistics, degrees of freedom and p-values were derived using maximum-likelihood-based deletion tests, following Crawley (2013).

**Normocytes**	
**Significant terms in minimal model**	
PHZ	$F(2, 65) = 282.38$, $p < 0.001$
**Non-significant terms deleted from model**	
Genotype	$F(3, 62) = 0.68$, $p = 0.567$
PHZ x Genotype	$F(6, 56) = 1.09$, $p = 0.381$
**Reticulocytes**	
**Significant terms in minimal model**	
PHZ	$F(2, 65) = 231.87$, $p < 0.001$
**Non-significant terms deleted from model**	
Genotype	$F(3, 62) = 2.48$, $p = 0.069$
PHZ x Genotype	$F(6, 56) = 2.19$, $p = 0.057$
**Frequency**	
**Significant terms in minimal model**	
PHZ	$F(2, 65) = 450.23$, $p < 0.001$
**Non-significant terms deleted from model**	
Genotype	$F(3, 62) = 0.75$, $p = 0.526$
PHZ x Genotype	$F(6, 56) = 1.31$, $p = 0.269$

Table S2. Summary of statistical model of cumulative asexual parasite growth (day 1 and 2 PI) by parasite genotype and PHZ treatment. The model was minimised and all test-statistics, degrees of freedom and p-values were derived using maximum-likelihood-based deletion tests, following Crawley (2013).

**Asexuals**	
**Non-significant terms deleted from model**	
PHZ	$F(2,65) = 2.81$, $p = 0.067$
Genotype	$F(3,62) = 1.62$, $p = 0.193$
PHZ x Genotype	$F(6,56) = 1.73$, $p = 0.132$

Table S3. Summary of statistical model of how conversion varies by PHZ treatment, parasite genotype and asexual density, using gametocyte density as a proxy for conversion. The model was minimised and all test-statistics, degrees of freedom and p-values were derived using maximum-likelihood-based deletion tests, following Crawley (2013).

**Gametocytes** R^2=0.84, df=51	
**Significant terms in minimal model**	
PHZ x Genotype	$F(6,51) = 4.34$, $p = 0.001$
Asexuals	$F(1,51) = 4.20$, $p = 0.046$
**Non-significant terms deleted from model**	
PHZ x Asexuals x Genotype	$F(6,40) = 0.22$, $p = 0.968$
Genotype x Asexuals	$F(3,46) = 0.34$, $p = 0.799$
PHZ x Asexuals	$F(2,49) = 0.50$, $p = 0.610$

Table S4. Summary of statistical models of how conversion varies with within-host physiological variables, using gametocyte density as a proxy for conversion. The proportion of reticulocytes (frequency) and total red blood cell density are the focus of model 1, reticulocyte density (model 2), normocyte density (model 3), and total red blood cell density (model 4). For each model, the density of asexuals was also controlled for. All models were minimised and all test-statistics, degrees of freedom and p-values were derived using maximum-likelihood-based deletion tests, following Crawley (2013).

**Model 1**	
**Significant terms in minimal model**	
Genotype x Frequency	$F(3, 56) = 7.50$, $p < 0.001$
**Non-significant terms deleted from model**	
Genotype x Frequency x Total Density x Asexuals	$F(3, 32) = 0.55$, $p = 0.652$
Frequency x Total Density x Asexuals	$F(1, 35) = 0.05$, $p = 0.828$
Genotype x Total Density x Asexuals	$F(3, 36) = 0.22$, $p = 0.880$
Genotype x Frequency x Asexuals	$F(3, 39) = 0.31$, $p = 0.820$
Genotype x Frequency x Total Density	$F(3, 42) = 0.49$, $p = 0.689$
TotalDensity x Asexuals	$F(1, 45) = 1.46$, $p = 0.232$
Frequency x Asexuals	$F(1, 46) = 0.28$, $p = 0.601$
Genotype x Asexuals	$F(3, 47) = 0.18$, $p = 0.911$
Frequency x Total Density	$F(1, 50) = 0.74$, $p = 0.393$
Genotype x Total Density	$F(3, 51) = 0.39$, $p = 0.761$
Asexuals	$F(1, 54) = 2.14$, $p = 0.149$
Total Density	$F(1, 55) = 0.03$, $p = 0.869$
**Model 2**	
**Significant terms in minimal model**	
Reticulocytes x Genotype	$F(3, 56) = 3.74$, $p = 0.016$
**Non-significant terms deleted from model**	
Genotype x Reticulocytes x Asexuals	$F(3, 48) = 0.93$, $p = 0.433$
Reticulocytes x Asexuals	$F(1, 51) = 0.03$, $p = 0.856$
Genotype x Asexuals	$F(3, 52) = 0.98$, $p = 0.407$
Asexuals	$F(1, 55) = 0.00$, $p = 0.999$

Model 3	
**Significant terms in minimal model**	
Normocytes x Genotypes	$F(3,55) = 6.54$, $p < 0.001$
Asexuals	$F(1,55) = 6.17$, $p = 0.016$
**Non-significant terms deleted from model**	
Genotype x Normocytes x Asexuals	$F(3,48) = 0.10$, $p = 0.958$
Normocytes x Asexuals	$F(1,51) = 0.40$, $p = 0.530$
Genotype x Asexuals	$F(3,52) = 0.48$, $p = 0.696$

Model 4	
**Significant terms in minimal model**	
Genotype x Total Density	$F(3,55) = 5.27$, $p = 0.003$
Asexuals	$F(1,55) = 9.09$, $p = 0.004$
**Non-significant terms deleted from model**	
Genotype x Total Density x Asexuals	$F(3,48) = 0.13$, $p = 0.943$
Total Density x Asexuals	$F(1,51) = 0.56$, $p = 0.459$
Genotype x Asexuals	$F(3,52) = 0.91$, $p = 0.440$

Table S5. Comparison of the minimal models described in table S4, ranked by AIC from lowest to highest.

Minimal Model	DF	R^2	AIC
**Model 1**			
Gametocytes ∼ Frequency + Genotype + Frequency x Genotype	56	0.81	1830.9
**Model 3**			
Gametocytes ∼ Normocytes + Genotype + Normocytes x Genotype + Asexuals	55	0.79	1840.0
**Model 4**			
Gametocytes ∼ Total Density + Genotype + Total Density x Genotype + Asexuals	55	0.74	1844.9
**Model 2**			
Gametocytes ∼ Reticulocytes + Genotype + Reticulocytes x Genotype	56	0.69	1867.6
